# Clinical characteristics and prognosis of elderly nasopharyngeal carcinoma patients receiving intensity-modulated radiotherapy

**DOI:** 10.1007/s00405-020-06399-5

**Published:** 2020-10-06

**Authors:** Yingchen Lyu, Mengshan Ni, Ruiping Zhai, Fangfang Kong, Chengrun Du, Chaosu Hu, Hongmei Ying

**Affiliations:** 1 Department of Radiation Oncology, Fudan University Shanghai Cancer Center, 270 Dong’an Road, Shanghai, 200032 China; 2 Department of Oncology, Shanghai Medical College, Fudan University, Shanghai, China

**Keywords:** Age-adjusted Charlson comorbidity index, Nasopharyngeal carcinoma, Intensity-modulated radiotherapy, Elderly

## Abstract

**Purpose:**

To evaluate the clinical characteristics and prognosis of elderly nasopharyngeal carcinoma (NPC) patients receiving intensity-modulated radiotherapy (IMRT).

**Methods:**

From June 2008 to October 2014, 148 newly diagnosed non-metastatic elderly NPC patients (aged ≥ 70 years) receiving IMRT were recruited. Comorbid condition was evaluated using the age-adjusted Charlson Comorbidity Index (ACCI). Kaplan–Meier method was used to estimate survival rates and the differences were compared using log-rank test. Hazard ratio (HR) and the associated 95% confidence interval (CI) were calculated using Cox proportional hazard model by means of multivariate analysis.

**Results:**

The median follow-up time was 66.35 months. Estimated OS rate at 5 years for the entire group was 61.8% (95% confidence interval [CI] 0.542–0.703). The 5-year OS rate of RT alone group was 58.4% (95% [CI] 0.490–0.696) compared with 65.2% (95% [CI] 0.534–0.796) in CRT group (*p* = 0.45).

In patients receiving IMRT only, ACCI score equal to 3 was correlated with superior 5-year OS rate in comparison with higher ACCI score 62.1% (95% [CI] 0.510–0.766) to 48.5% (95% [CI] 0.341–0.689), respectively; *p* = 0.024). A 5-year OS rate of 63.1% (95% [CI] 0.537–0.741) was observed in patients younger than 75 years old compared with 57.5% (95% [CI] 0.457–0.723) in patients older (*p* = 0.026). Patients with early-stage disease (I–II) showed better prognosis than patients with advanced-stage (III–IV) disease (5-year OS, 72.3–55.4%, respectively; *p* = 0.0073). The Cox proportional hazards model suggested that age independently predicted poorer OS (HR, 1.07; 95%CI 1.00–1.15, *p* = 0.04).

**Conclusion:**

The survival outcome of patients aged ≥ 70 years receiving IMRT only was similar to chemoradiotherapy with significantly less acute toxicities. Among the population, age is significantly prognostic for survival outcomes.

## Introduction

Nasopharyngeal carcinoma is characterized by its unique and extremely unbalanced geographical distribution, with 70% cases in the east and Southeast Asia [1]. Unlike the bimodal distribution in low-risk populations, the age distribution in epidemic area peaks in individuals aged 45–59 [2, 3]. As the percentage of elderly people is gradually increasing globally, the occurrence of NPC in the elderly is not rare. Despite the heavier health burden of the geriatric population, elderly individuals have been underrepresented in clinical studies. Particularly, those aged ≥ 70 years attributed less than 5% in prospective trials in head and neck cancer [4]. In MAC-NPC meta-analysis, those aged ≥ 60 years constituted merely 13% of the total cohort [5]. The proportion of ≥ 70 years age was likely less than half of that figure. The treatment paradigms in the elderly NPC patients have not been well defined for the limited researches.

Opinions were divided on whether standard aggressive treatment can bring benefit to this vulnerable population. Some argued that elderly NPC patients should receive chemotherapy for improved survival outcome [6–8]. In those mentioned studies, most of the patients were treated with conventional radiotherapy. It was not clear that adding chemotherapy could bring benefit to patients receiving IMRT, which has now been widely used with excellent locoregional control in NPC. Moreover, selecting 60–65 as the cut-off value in those studies for the elderly may not be reasonable for the improved physical health of this population. Patients aged ≥ 70 years were closely associated with multiple comorbidities, poor performance status and reduced organ reserve, which accounted for their lower tolerance and severe toxicity for chemotherapy [9, 10]. Thus, tailored and less aggressive treatment strategies in elderly individuals should be considered.

In this article, we focused on the survival and prognosis of senior NPC patients receiving Intensity-Modulated radiotherapy and assessed the comorbidities utilizing age-adjusted Charlson Comorbidity Index.

## Materials and methods

### Patient characteristics

This retrospective study received approval from the Institutional Review Board. The document requirement was waived because of the retrospective nature of the study. The research presented no more than minimal risk with only clinical and dosimetric data studied.

Between June 2008 and October 2014, a total of 148 NPC patients undergoing IMRT at Fudan University Shanghai Cancer Center (FUSCC) were included in this study. The eligible criteria were: (1) age of 70 years old and above; (2) histologically proven NPC; (3) treated with IMRT. The exclusion criteria were: (1) presence of distant metastasis; (2) underwent surgery; (3) history of head and/or neck irradiation; (4) Karnofsky Performance Score < 70.

### Clinical staging and co-morbidity assessment

Pretreatment assessment consisted of complete patient history, thorough physical examination, hematology and biochemistry profiles, nasopharyngeal magnetic resonance imaging (MRI), neck MRI or computed tomography (CT), bone scan, chest X-ray or CT, abdominal ultrasound. All patients underwent restaging according to the eighth edition guideline of the American Joint Commission on Cancer staging. Considering the vulnerable nature of the studied population, comorbid conditions were evaluated using the age-adjusted Charlson Comorbidity Index (ACCI), which has been shown to be an independent predictor of long-term survival.

### Radiotherapy

Patients were immobilized in the supine position with a thermoplastic mask. CT was performed after immobilization, obtaining 5-mm slices from the anterior clinoid process 2 cm below the sternoclavicular joint. All the patients received IMRT with six megavoltage photons (6MV). In brief, the total dose was 66–70.4 Gy in 30–35 fractions to primary lesion of nasopharynx, 66 or 70.4 Gy in 30–35 fractions to metastatic lymph nodes of the neck, 60 Gy to high-risk CTV and 54 Gy to low-risk CTV, respectively. Small-field IMRT was applied to treat local residual disease just after the planned treatment with 2.2–4.4 Gy in one or two fractions. Residual nodes were treated with a boost of 4–6 Gy in 2 or 3 daily fractions using an electron field of 9–12 meV just after the planned treatment.

### Follow-up and statistical analysis

During radiotherapy, patients underwent assessment weekly. After completing radiotherapy, patients were followed up every 3 months in the first 2 years, and then every 6 months from year 2 to year 5, and annually thereafter. Survival time was measured from the initiation of the RT to date of death or the latest date of follow-up for patients still alive.

Kaplan–Meier method was used to estimate survival rates and the differences were compared using log-rank test. Hazard ratio (HR) and the associated 95% confidence interval (CI) were calculated using Cox proportional hazard model. The *χ*^2^ test was used for comparing categorical variables, and independent *t*-test was used for comparing the means of continuous variables. Covariates including age, sex, age-adjusted Charlson Comorbidity Index, *N* category, *T* category, overall stage, radiation dose, and chemotherapy were included in all tests. Cox proportional hazards model was performed to carry out univariate analysis and multivariate analysis. A two-sided *p *value of < 0.05 was considered statistically significant. All data analyses and drawings were completed using R software 4.0.0 (https://www.r-project.org).

## Results

### Baseline characteristics

Table 1 demonstrated the patients’ characteristics. Median age was 74.18 years (age ranged 70–93 years). The ratio of male to female was 3.48:1, with 115 males and 33 females. According to the AJCC/UICC (8th edition) staging criteria, there were 26 (17.6%) patients with stage I disease, 53 (35.8%) patients with stage II disease, 31 (20.9%) patients with stage III disease and 28 (18.9%) patients with stage IV disease. The comorbidity level was sored according to the ACCI. The score was 3 in 105 (70.9%), 4 in 27 (18.2%), 5 in 12 (8.1%), 6 in 2 (1.4%), 7 in 1 (0.7%) and 13 in 1 (0.7%). 89 patients received IMRT only. Induction chemotherapy was applied to 44 (27.2%) with concurrent chemotherapy in 27 (18.2%) and adjuvant chemotherapy in 6 (4.1%). Patients receiving radiotherapy alone tended to have older age (median age 75.24 vs.72.58) (*p* < 0.001), earlier stage (*p* < 0.001) and higher ACCI (Table 2).

**Table 1:** Baseline characteristics of patients

Level	No. of patients
*n*	148
Age [mean (SD)]	74.18 (3.86)
Gender (%)	
Female	33 (22.3)
Male	115 (77.7)
ACCI (%)	
3	105 (70.9)
4	27 (18.2)
5	12 (8.1)
6	2 (1.4)
7	1 (0.7)
13	1 (0.7)
Treatment (%)	
RT alone	89 (60.1)
Induction CT	44 (27.2)
Concurrent CT	27 (18.2)
Adjuvant CT	6 (4.1)
*T* (%)	
1	26 (17.6)
2	53 (35.8)
3	31 (20.9)
4	38 (25.7)
*N* (%)	
0	28 (18.9)
1	67 (45.3)
2	39 (26.4)
3	14 (9.5)
8th AJCC stage (%)	
1	11 (7.4)
2	37 (25.0)
3	52 (35.1)
4	48 (32.4)
Radiation dose (%)	
≤ 6600 Gy	75 (50.7)
> 6600 Gy	73 (49.3)
Follow-up time (months) (median [IQR])	66.35 [29.03, 86.74]

*ACCI* age-adjusted Charlson Comorbidity Index, *AJCC* American Joint Commission on Cancer staging, *CT* chemotherapy, *IQR* Inter-Quartile Range

**Table 2:** Comparation of baseline characteristics in RT alone and CRT group

Level	RT	CRT	*p*
*n*	89	59	
Age [mean (SD)]	75.24 (4.21)	72.58 (2.59)	** < 0.001**
Gender (%)			
0	22 (24.7)	11 (18.6)	0.426
1	67 (75.3)	48 (81.4)	
ACCI (%)			
3	56 (62.9)	49 (83.1)	0.117
4	20 (22.5)	7 (11.9)	
5	9 (10.1)	3 (5.1)	
6	2 (2.2)	0 (0.0)	
7	1 (1.1)	0 (0.0)	
13	1 (1.1)	0 (0.0)	
*T* (%)			
1	23 (25.8)	3 (5.1)	**0.001**
2	34 (38.2)	19 (32.2)	
3	15 (16.9)	16 (27.1)	
4	17 (19.1)	21 (35.6)	
*N* (%)			
0	23 (25.8)	5 (8.5)	**0.001**
1	45 (50.6)	22 (37.3)	
2	16 (18.0)	23 (39.0)	
3	5 (5.6)	9 (15.3)	
8th AJCC stage (%)			
1	11 (12.4)	0 (0.0)	** < 0.001**
2	31 (34.8)	6 (10.2)	
3	25 (28.1)	27 (45.8)	
4	22 (24.7)	26 (44.1)	
Radiation dose [mean (SD)]	6700.90 (431.97)	6885.07 (211.52)	**0.003**
Follow-up time (months) (median [IQR])	70.53 [28.63, 95.40]	63.03 [31.30, 80.93]	0.357

Bold values denote statistical significance at the *p* < 0.05 level

*p *value was calculated using the Pearson *χ*^2^ test or Fisher’s exact test

*CRT *chemoradiotherapy, *RT *radiotherapy

### Radiotherapy course and toxicity

In all, 144 patients (97.3%) completed the planned RT. Of four who failed, one patient received the total dose < 60 Gy because of treatment toxicities, two patients were administrated with 60 Gy because of their own will. The median actuarial irradiated dose of the nasopharynx was 66 Gy (range 35.2–74.4 Gy), and 10 patients (6.7%) received a boost for residual primary disease.

The effect of acute toxicity in radiotherapy (RT) alone and chemoradiotherapy (CRT) group was shown in Table 3. The rates of severe mucositis and dermatitis were higher in CRT group. Besides, CRT group developed significantly higher leukopenia, Neutrocytopenia, Thrombocytopaenia (*p* < 0.001) compared to RT alone group. In RT alone group, the majority developed with grade 1 to 2 toxicity. Severe acute mucositis and dermatitis (grade 3 or 4) occurred in 24.72% and 5.62%, respectively. Emesis occurred in 11.2% of patients without grade 3 or 4 events. For hematologic adverse events, incidence of grade 3 leukopenia was 1.12%, which was the same with incidence of grade 3 neutrocytopenia and thrombocytopenia. No grade 4 hematologic toxicity was observed. Details of the acute toxicity are listed as Table 3.

**Table 3:** Acute toxicities in RT alone and CRT group

Level	RT	CRT	*p* value
*N* (no. of patients)	89	59	
Mucositis (%)			
0	19 (21.3)	11 (18.6)	0.477
1	19 (21.3)	10 (16.9)	
2	29 (32.6)	15 (25.4)	
3	20 (22.5)	21 (35.6)	
4	2 (2.2)	2 (3.4)	
Dermatitis (%)			
0	49 (55.1)	33 (55.9)	0.267
1	18 (20.2)	14 (23.7)	
2	17 (19.1)	6 (10.2)	
3	3 (3.4)	1 (1.7)	
4	2 (2.2)	5 (8.5)	
Emesis (%)			
0	79 (88.8)	53 (89.8)	0.847
1	5 (5.6)	2 (3.4)	
2	5 (5.6)	4 (6.8)	
Leukopenia (%)			
0	72 (80.9)	30 (50.8)	** < 0.001**
1	11 (12.4)	8 (13.6)	
2	5 (5.6)	8 (13.6)	
3	1 (1.1)	12 (20.3)	
4	0 (0.0)	1 (1.7)	
Neutrocytopenia (%)			
0	85 (95.5)	32 (54.2)	** < 0.001**
1	0 (0.0)	4 (6.8)	
2	3 (3.4)	11 (18.6)	
3	1 (1.1)	9 (15.3)	
4	0 (0.0)	3 (5.1)	
Thrombocytopaenia (%)			
0	81 (91.0)	38 (64.4)	**0.001**
1	4 (4.5)	10 (16.9)	
2	3 (3.4)	6 (10.2)	
3	1 (1.1)	4 (6.8)	
4	0 (0.0)	1 (1.7)	
Anemia (%)			
0	82 (92.1)	51 (86.4)	0.32
1	7 (7.9)	6 (10.2)	
2	0 (0.0)	1 (1.7)	
3	0 (0.0)	1 (1.7)	

Bold values denote statistical significance at the *p* < 0.05 level

*p *value was calculated using the Pearson *χ*^2^ test or Fisher’s exact test

*CRT* chemoradiotherapy, *RT *radiotherapy

### Prognostic factors and survival

**Fig. 1:**
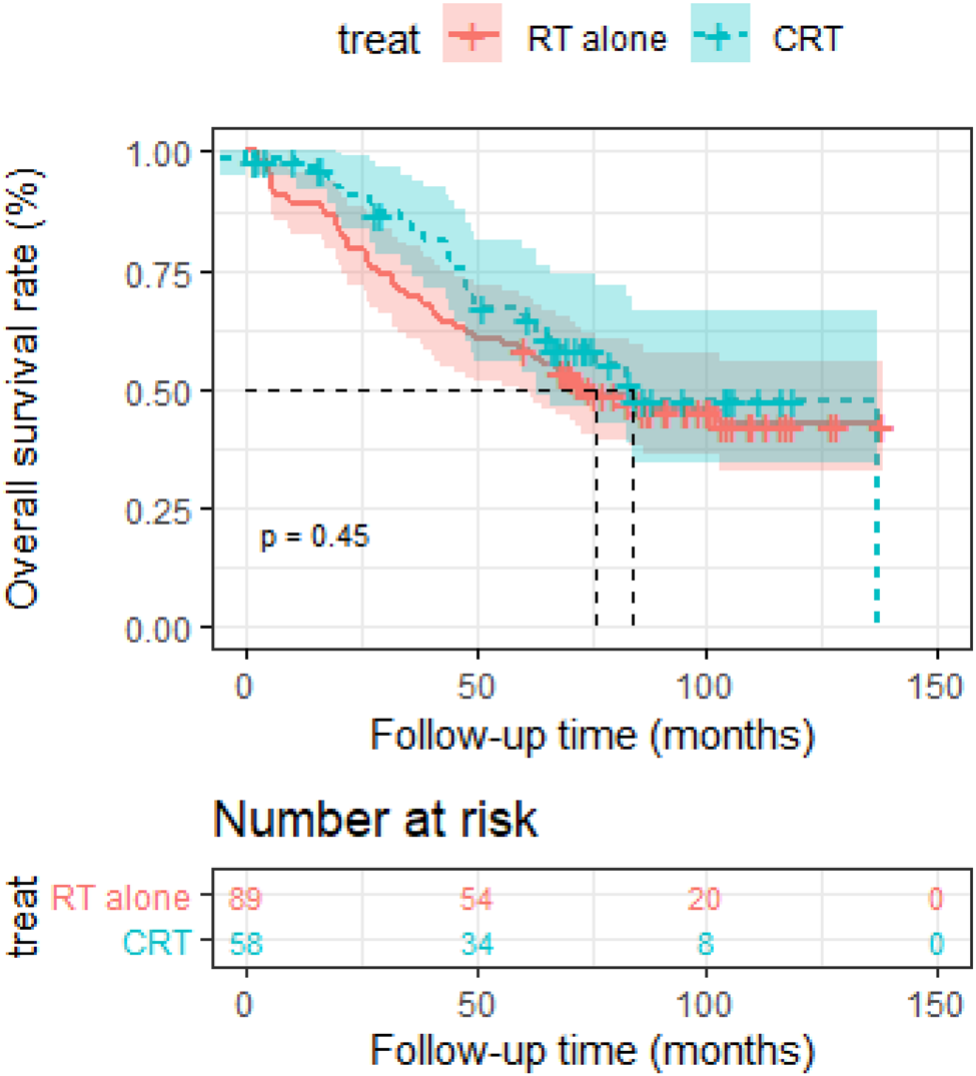
Kaplan–Meier survival curves between the RT and CRT groups in elderly patients. Shown in results in overall survival (OS). *P* values were calculated using the unadjusted log-rank test

The median follow-up time was 66.35 months. Estimated OS rate at 5 years for the entire group was 61.8% (95% confidence interval [CI] 0.542–0.703). The addition of chemotherapy to IMRT failed to enhance the survival outcomes of patients. The 5-year OS rate of RT alone group was 58.4% (95% [CI] 0.490–0.696) compared with 65.2% (95% [CI] 0.534–0.796) in CRT group (*p* = 0.45) (Fig. 1).

**Fig. 2:**
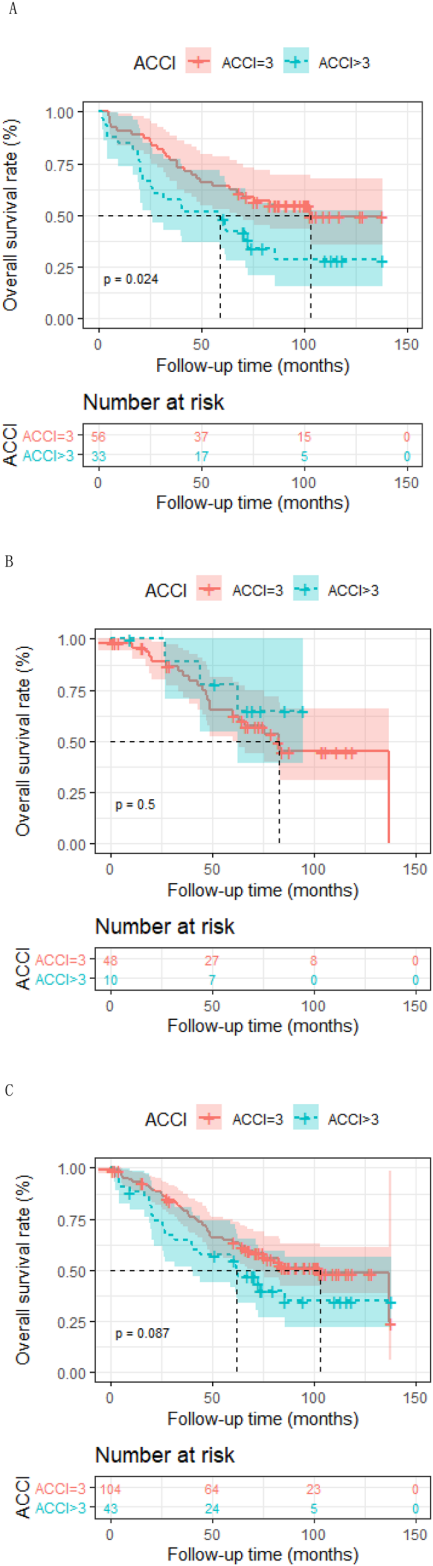
Kaplan–Meier analysis of overall survival is stratified by ACCI in RT alone group (**a**), CRT group (**b**) and the whole group (**c**). ACCI, age-adjusted Charlson Comorbidity Index

**Fig. 3:**
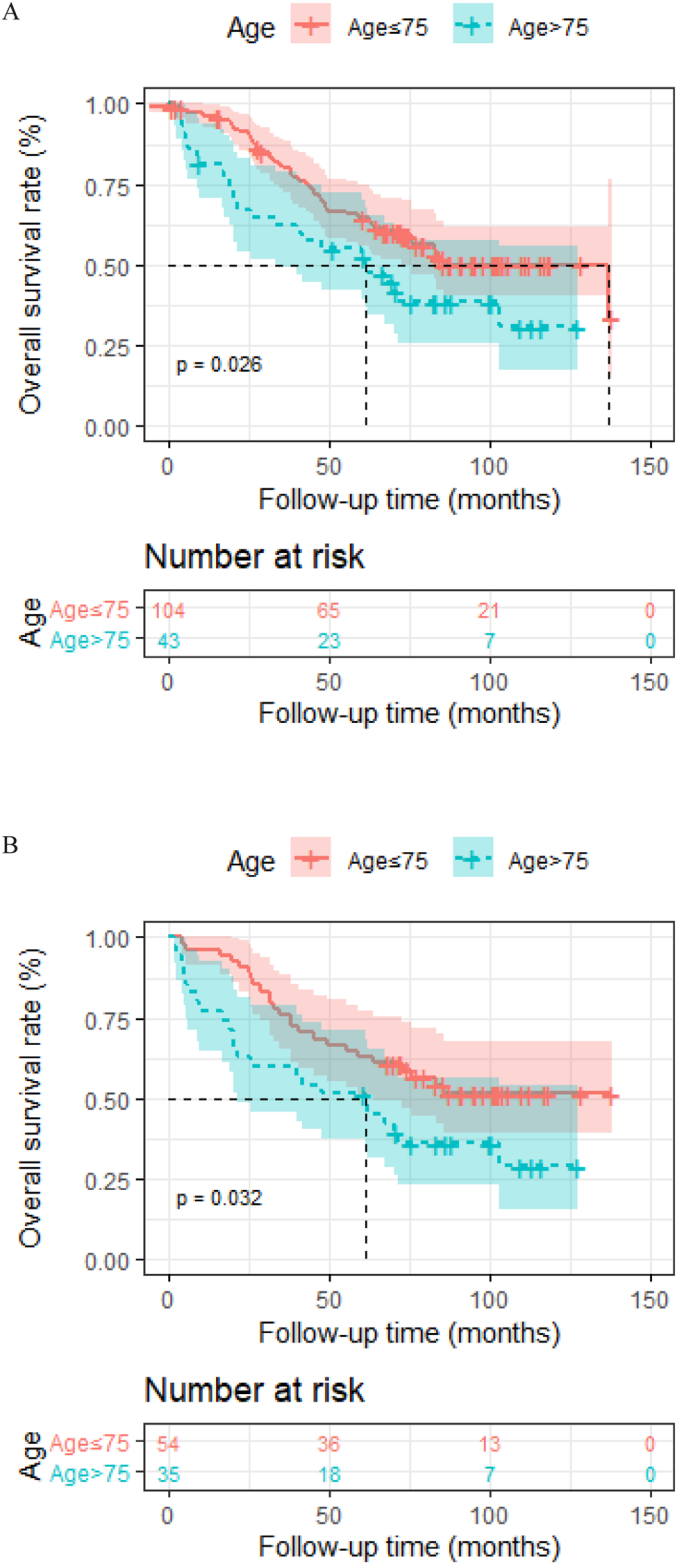
Kaplan–Meier analysis of overall survival is stratified by age in the entire group (**a**) and RT alone group (**b**)

**Fig. 4:**
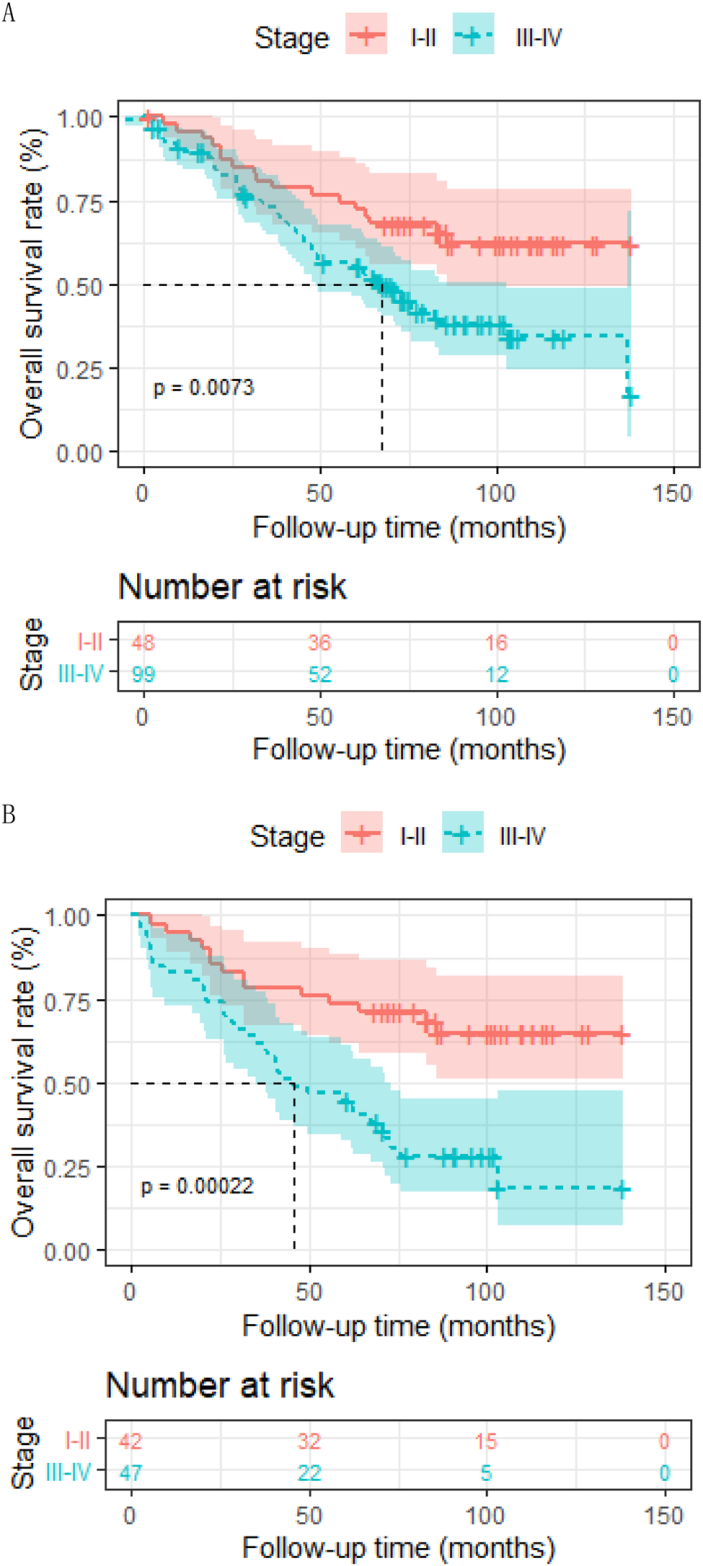
Kaplan–Meier analysis of overall survival is stratified by stage in the entire group (**a**) and RT alone group (**b**)

In patients receiving IMRT only, ACCI score equal to 3 was correlated with superior 5-year OS rate in comparison with higher ACCI score 62.1% (95% [CI] 0.510–0.766) to 48.5% (95% [CI] 0.341–0.689), respectively; *p* = 0.024) (Fig. 2a). A 5-year OS rate of 63.1% (95% [CI] 0.537–0.741) was observed in patients younger than 75 years old compared with 57.5% (95% [CI] 0.457–0.723) in patients older (*p* = 0.026) (Fig. 3a). In patients receiving RT only, the overall survival of aged ≤ 75 was also superior to the elder counterpart (*p* = 0.032) (Fig. 3b). Patients with early-stage disease (I–II) showed better prognosis than patients with advanced-stage (III–IV) disease (5-year OS, 72.3% to 55.4%, respectively; *p* = 0.0073) (Fig. 4a). In patients receiving RT only, prognosis of early-stage was also more favorable than advanced-stage diseases (*p* = 0.00022) (Fig. 4b). However, the positive association of radiation dose with OS in both groups was not found.

The Cox proportional hazards model (Table 4) suggested that age independently predicted poorer OS (HR, 1.07; 95%CI 1.00–1.15, *p* = 0.04). Besides, ACCI score higher than 3, tumor stage and clinical stage were associated with poor prognosis in univariate analysis but were not significantly independent prognostic predictors in multivariate analysis.

**Table 4:** Cox proportional hazard model

Variable	Univariate analysis		Multivariate analysis	
	HR (95%CI)	*p *value	HR (95%CI)	*p *value
Age	1.09 (1.02–1.16)	**0.01**	1.07 (1.00–1.15)	**0.04**
Sex (male vs. female)	1.22 (0.69–2.15)	0.499	NS	NS
T category (T1-2 vs. T3-4)	1.7 (1.07–2.7)	**0.026**	1.29 (0.72–2.32)	0.39
N category (N0-1 vs. N2-3)	1.44 (0.9–2.31)	0.131	NS	NS
Overall stage (I–Ii vs. III–IV)	2.08 (1.2–3.59)	**0.009**	1.79 (0.90–3.56)	0.10
Radiation dose(≤ 6600 vs. > 6600)	1.38 (0.87–2.19)	0.175	NS	NS
ACCI score (≤ 3 vs. > 3)	1.52 (0.94–2.47)	0.090	1.46 (0.86–2.48)	0.16
RT alone vs. CRT	0.83 (0.51–1.35)	0.450	NS	NS

*ACCI* age-adjusted Charlson Comorbidity Index, *AJCC* American Joint Commission on Cancer staging, *HR* hazard ratio, *CI* confidence interval, *NS* not significant, *CRT *chemoradiotherapy, *RT *radiotherapy

## Discussion

It should be noted that elderly NPC patients have been underrepresented in clinical studies for restrict inclusion criteria. Accompanied with poor performance status, multiple comorbidities and decreasing organ function, they tended to be excluded from clinical trials. In MAC meta-analysis, patients older than 60 years old accounted for merely 13%. Moreover, patients older than 70 years old were estimated less than half of that figure [5]. Treatment guidelines were principally tailored for non-elderly patients. Concurrent chemoradiotherapy (CCRT) has been accepted as standard treatment modalities for locally advanced NPC. Nevertheless, it remained unknown whether elderly NPC patients can benefit from chemotherapy while the adding of chemotherapy carries significantly higher rates of acute toxicity. A Meta-analysis of Chemotherapy in Head and Neck Cancer (MACH-NC) found that concomitant chemotherapy brought an absolute benefit of 6.5% at 5 years and 9.3% in local control rate, though the effect of chemotherapy decreased with increasing age (*p* = 0.003). Patients aged > 70 failed to benefit from concomitant chemotherapy [11].

The cut-off age defining elderly patients varied in different studies. Developed countries have accepted the chronological age of 65 and older as a definition of an elderly population. The National Institute on Aging at the National Institutes of Health classify elderly patients into three categories: young old (65–74 years), older old (75–85 years), and oldest old (> 85 years) [12]. With improved physical health of patients, some people who were defined as “old” might be able to withstand aggressive treatment and have favorable prognosis. Patients aged ≥ 70 years were closely associated with multiple comorbidities, poor performance status and reduced organ reserve, which accounted for their lower tolerance and severe toxicity for chemotherapy [9, 10]. The reason for choosing 70 as the cut-off points for elderly was that it was close to the threshold in MACH-NC study [11]. Besides, it is often used in head and neck cancer and other type of cancers [13].

In our data, patients receiving radiotherapy alone tended to have older age and more comorbidities. Besides, patients with abnormal hematological abnormalities were less likely to receive chemotherapy at our center considering more treatment-related toxicities. As shown in our data, hematological adverse effects including leukopenia, neutrocytopenia and thrombocytopaenia were significantly higher in CRT group. Elderly patients with comorbidities may be more prone to have severe toxicities, suffer from treatment delays or dose reductions, therefore, they may not survive long enough to derive expected benefits from chemotherapy [14].

Two studies demonstrated that chemotherapy improved survival outcome for elderly NPC patients with conventional 2DRT. The 5-year OS rates of the RT alone and chemoradiotherapy groups in NPC patients aged ≥ 60 years reported by Zeng et al. [6] were 40% and 62% (*p* = 0.013). Liu et al. made similar conclusion in patients aged > 60 years receiving conventional radiotherapy [10]. Nonetheless, it was doubted that chemotherapy could bring benefit to geriatric NPC patients with widespread use of IMRT, which has obtained great local control, better protection for normal organs. In the era of IMRT, whether adding chemotherapy could still benefit elderly individuals remained uncertain. Our data suggested that addition of chemotherapy to IMRT failed to enhance the survival outcomes of patients. The 5-year OS rate of RT alone group was 58.4% (95% [CI] 0.490–0.696) compared with 65.2% (95% [CI] 0.534–0.796) in CRT group (*p* = 0.45).The survival outcome was comparable to Jin et al.’s study, which failed to confirm the benefit of chemotherapy for elderly NPC patients older than 70 years (5-year OS 56.6–51.2%, *p* = 0.617). Moreover, in patients with Adult Comorbidity Evaluation-27 Index (ACE-27) ≥ 2, chemotherapy was associated with an even inferior OS (26.4% vs. 50.3%, *p* = 0.071) [15]. In Sommat et al.’s [16] study, chemotherapy did not bring benefit in stage III and IVA/B patients (aged ≥ 70). In the PSM cohort of Wen et al.’s [17] study (patients over 70 years old), the 3-year CSS was similar in the RT group compared to CRT group (64.3% vs. 65.2%, *p* = 0.764). There was no significant difference in other clinical endpoints assessed, whether chemoradiotherapy could bring benefit to elderly patients with other head and neck cancers worth considering. Recently, Erik Haehl et al. [18] suggested that addition of chemotherapy resulted in a survival benefit for patients aged 65–74 years in the definitive but not in the adjuvant treatment cohort. However, it should be noted that only 4 NPC patients were included in their study group. Besides, the age-dependent outcome in elderly HNSCC patients were similar to our finding that age was an independent prognostic factor.

In terms of toxicity, the incidence rate for severe mucositis and dermatitis (grade 3–4) was 24.7% and 5.6% in RT alone group compared to 39% and 10.2% in CRT group (*p* < 0.001). Yang et al. [7] suggested severe acute mucositis rates of 16.8% and 39.8% in IMRT-alone and IMRT plus CCRT groups (*p* < 0.001). Jin et al. [15] reported an incidence of 18.3% grade 3–4 mucositis in the entire group. In the PSM cohort of Wen et al.’s [17] study, there was no severe neutropenia and emesis in IMRT-alone group, which was significantly lower than in CCRT group. Thus, IMRT greatly reduced the incidence of severe acute toxicities and increased tolerance to treatment.

Noteworthy, in patient receiving RT only, ACCI score equal to 3 was correlated with superior 5-year OS rate in comparison with higher ACCI score 62.1% (95% [CI] 0.510–0.766) to 48.5% (95% [CI] 0.341–0.689), respectively; *p* = 0.024). ACCI was found to be a prognostic factor for OS in our cohort in the univariate, but not the multivariate analysis. Among the methods quantifying the comorbid ailments, Charlson comorbidity index was used most extensively for its simplicity and straightforward nature. Researches suggested that presence, type and severity of comorbidity condition had an impact on the treatment selection and survival outcome in aged head and neck cancer patients [19, 20]. Acknowledging the relationship between age and comorbidity in cancer, studies suggest that comorbidity increases with age among head and neck populations [21, 22]. Due to the importance of age on comorbidity, ACCI, the modified CCI, includes age of the patients as correction variable [23]. A population-based study of 4095 NPC patients in Taiwan found that overall survival was significantly associated with the degree of comorbidity and ACCI was a more appropriate prognostic indicator than either original CCI or a revised head and neck comorbidity index (HN-CCI) [24]. Besides, accessing comorbidity level provided information independent from personal performance status. A study of 203 aged cancer patients revealed that the comorbidity condition was not related to performance status [25].

Previous studies reported poor survival for the elderly compared with the younger counterpart [9, 26, 27]. Comorbidity level, DNA copies and other factors, such as CRP and hemoglobin, have been studied for their prognostic value [7, 28]. More potential biomarkers should be studied to select patients who might be fit for chemoradiotherapy. Biological age other than chronological age should be the dominant factor in the selection of best treatment approach for patients with locoregionally confined head and neck cancer.

Integrating these factors into a comprehensive assessment tool might help guide personalized treatment decision and predict survival for elderly NPC patients. A study tried to combine comorbidity level and TNM staging to address this gap [21, 29]. Other factors, such as age, race, gender, socioeconomic status and functional status, might also shed light on clinical decision-making for both health providers and patients.

There are some limitations in our study. First, this study is limited by its retrospective nature, though the prospective trials for the elderly may be hindered by the rarity of patients and the prevalence of comorbidities and decreasing organ function. Second, physical activity assessment, such as Karnofsky performance status (PS), should be concerned, which had been identified as prognostic factor for other head and neck cancers but failed to show significant effect on survival of NPC [30, 31]. Besides, data on chronic toxicities and quality of life were missing in this study. In Jin et al.’s [15] study, four patients (3.2%) developed radiation-induced TLI. And our study did not discuss potential biomarkers, such as EBV-DNA, CRP or hemoglobin, which may help select patients for treatment [7, 28]. Another limitation of this study is the small sample size. With the low incidence of NPC in elderly population, this could be acceptable. Therefore, the treatment paradigm and prognostic factors for elderly NPC patients in the era of IMRT warrant further investigation.

In conclusion, our study presented that the survival outcome of patients aged ≥ 70 years receiving IMRT only was similar to chemoradiotherapy with significantly less acute toxicities. Among the population, age is significantly prognostic for survival outcomes. Further investigation is urgently needed to fully assess the elderly individuals and personalize treatment strategy.

## Data Availability

The datasets used and analyzed during the current study are available from the corresponding author on reasonable request.
